# A large gastrointestinal stromal tumor of the duodenum: a case report

**DOI:** 10.1186/1752-1947-5-457

**Published:** 2011-09-14

**Authors:** Basem Morcos, Firas Al-Ahmad

**Affiliations:** 1 Department of Surgical Oncology, King Hussein Cancer Center, Queen Rania Al Abdullah Street, P.O.Box 1269 Al-Jubeiha, Amman, 11941, Jordan

## Abstract

**Introduction:**

Gastrointestinal stromal tumors of the duodenum are uncommon. They can reach a large size. Diagnosis can be elusive and managing them can be difficult. Our case report aims to increase awareness and highlight some issues related to the diagnosis and management of duodenal gastrointestinal stromal tumors.

**Case presentation:**

We present the case of a 38-year-old Middle Eastern woman with a large, slowly-growing gastrointestinal stromal tumor of the duodenum. Her complaints were minor epigastric discomfort and swelling. A pancreaticoduodenectomy with complete tumor excision was performed. She was doing very well with no evidence of disease recurrence when she was last seen 34 months after her operation.

**Conclusion:**

Gastrointestinal stromal tumors of the duodenum should be suspected in any patient with a duodenal wall mass. Extramural growth and central ulceration with or without bleeding should alert the endoscopist to the possibility of a duodenal gastrointestinal stromal tumor diagnosis. There is more than one surgical approach available; however, complete surgical excision, with negative margins, is the absolute requirement. Preoperative imatinib mesylate can be considered in unresectable or borderline resectable cases.

## Introduction

The most common sites for gastrointestinal stromal tumors (GIST) are the stomach and, to a lesser extent, the small intestine [1]. Small intestinal GIST can occur anywhere along the length of the bowel and can be multiple. The duodenum is involved in about 10% to 20% of small intestinal GIST [2]. Although duodenal GIST is similar pathologically to that involving other organs, they do have some peculiar features. GISTs in the duodenum pose particular challenges for diagnosis and management.

We describe the case of a large duodenal GIST including its presentation, diagnosis, and the type of surgery performed, as well as a review of issues related to GIST in the duodenum.

## Case presentation

A 38-year-old Middle Eastern woman presented with a slowly enlarging abdominal mass of 12 years duration. According to the patient, a surgeon had attempted to resect the mass 12 years earlier, but could not do so due to excessive bleeding from the tumor. She was offered no further treatment.

At presentation, her main complaint was epigastric discomfort. She also gave a history of some mild back pain and occasional abdominal pain. Her appetite was good and she had not lost weight. There was no history of vomiting, change in bowel habits or melena. She had been diagnosed with a peptic ulcer many years ago.

**Figure 1:**
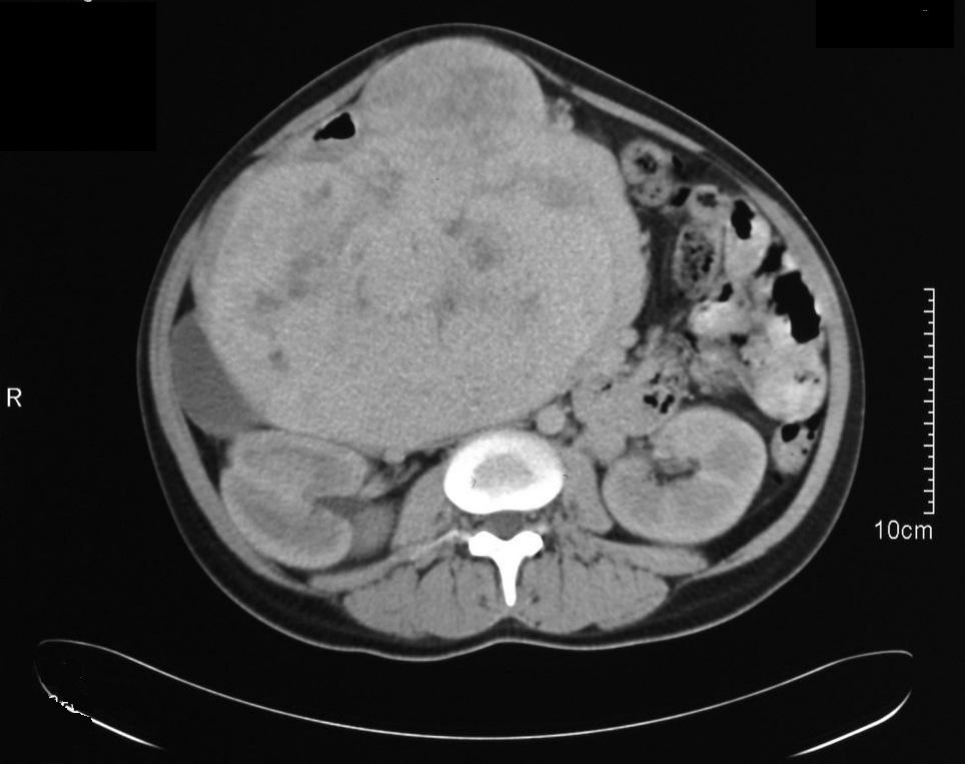
**Retropancreatic tumor**. A preoperative CT scan showing the large retropancreatic tumor.

On examination she looked healthy with no clinical jaundice or pallor. Abdominal examination revealed a large upper abdominal mass with thinned overlying skin. It had minimal mobility and was not tender. The rest of the examination was normal. Her hemoglobin level was 10.8 g/dL, with hypochromic microcytic red blood cell indices. Otherwise, all blood tests were normal. A computed tomography (CT) scan of the abdomen revealed a 20 cm retroperitoneal mass in the region of the head of the pancreas (Figure 1). It appeared to push and stretch the surrounding structures. There was no evidence of metastases to the liver or lung. Upper gastrointestinal endoscopy was performed, showing a 2.5 cm ulcer in the second part of the duodenum with a clot at its center. There was no intraluminal mass. A deep biopsy was taken, but was not diagnostic.

**Figure 2:**
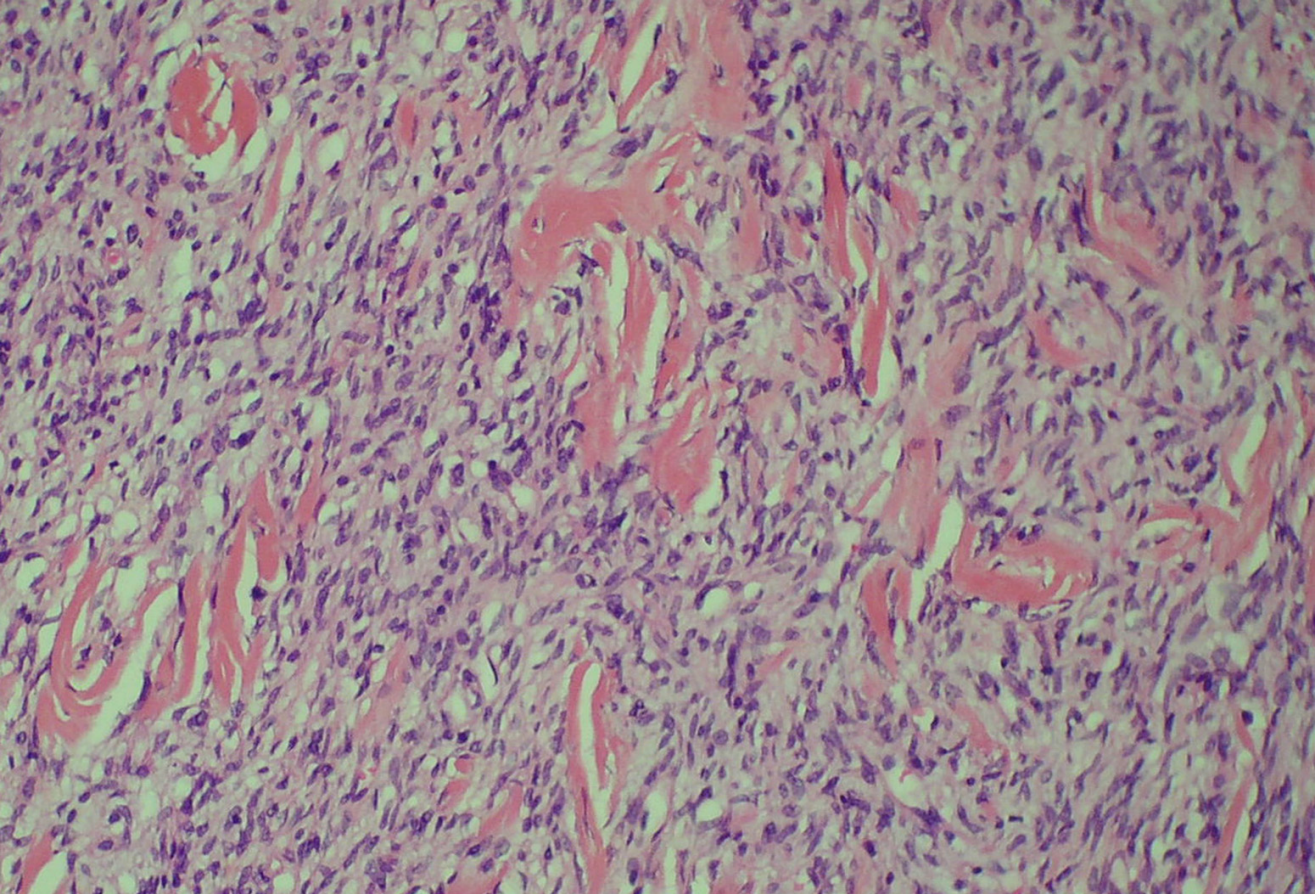
**Tumor histopathology**. Hematoxylin and eosin (H&E) slide. Notice the spindle cells with abundance of skeinoid fibers which are features of gastrointestinal stromal tumors.

Tumor embolization was planned to decrease tumor vascularity before resection. Angiography revealed that the hepatic artery was the main feeding vessel; however, embolization was not possible because the celiac axis was kinked and the catheter could not be advanced into the feeding artery. After preparation she was taken to the operating theater. A midline incision over the previous scar was performed. The tumor was very vascular with large venous tributaries draining into the portal circulation. It lay posterior to the pancreatic head and duodenum, pushing them anteriorly. A pancreaticoduodenectomy (Whipple procedure) was performed with the dissection kept outside the pseudocapsule of the tumor, taking care not to rupture the tumor. The patient tolerated the procedure well and had an uneventful recovery. Histopathological examination revealed a 22 cm tumor arising from the second part of the duodenum. The tumor showed moderate cellularity and mildly atypical spindle cells arranged in fascicles with a low mitotic count (1/50 high power field) and no necrosis (Figure 2). Prominent skeinoid fibers were seen. The tumor was negative for c-kit, SMA and S100 protein, but positive for CD34. Although it was c-kit negative, the features were consistent with the diagnosis of GIST. The tumor was considered of high malignant potential because of its size. Imatinib mesylate (IM) was considered as an adjuvant treatment but the patient could not afford it. She continued to do well, however, and was free of any recurrence the last time she attended the clinic, 34 months after the operation.

## Discussion

GISTs are the most common mesenchymal tumors of the gastrointestinal tract [1]. They are most commonly found in the stomach and small bowel. Uncommon sites include the colon, rectum, esophagus and even the liver and mesentery. They mainly affect adults and are uncommon in children [3]. The duodenum is an uncommon site for GIST. It comprises 10%-20% of small-intestinal GISTs, or only three to five percent of all GIST cases [4]. Most data on duodenal GIST are from single case reports or from a few small series [4,5]. Duodenal GIST is usually asymptomatic when small in size and can reach a large size before causing any symptom. As the tumor enlarges it causes variable symptomatology. The most common presentation is gastrointestinal bleeding which may be chronic and mild or sudden and massive [6]. Although our patient had a large tumor, she had mild anemia. The next most common presentations are abdominal discomfort, pain and swelling [5].

Diagnosis can be made with upper gastrointestinal endoscopy [5]. The tumor is usually exophytic, and appears as a submucosal swelling. Sometimes it presents only as an ulcer, as in our case. The biopsy should be deep, but may not always be diagnostic. Endoscopic ultrasound can help in delineating the submucosal tumor. A CT scan of the abdomen usually shows a retroperitoneal tumor at the site of the duodenum and head of the pancreas [7]. However, CT scans are not always helpful in specifying the origin of the mass. In a number of cases reported in the literature, the mass was misdiagnosed as arising from the head of the pancreas [8].

The treatment of choice for duodenal GIST is complete surgical excision. This can be performed by local or segmental duodenal resection with preservation of the pancreas for small tumors [2]. As for larger tumors, a pancreaticoduodenectomy is required. The surgical choice depends not only on the size of the tumor but also on the location in the duodenal wall and the relation to the ampulla of Vater. It is not clear what the optimal surgical margin should be, but a negative one is essential to prevent local recurrence of the tumor. No lymph node dissection is required since they are very unlikely to be involved [1].

The outcome depends on the pathological features of the tumor and the completeness of surgical resection. Large tumors with high mitotic counts behave much worse than small tumors with low mitotic counts, which are considered benign [9]. Local recurrence is higher in tumors not completely removed or with a positive microscopic margin. Most GISTs respond to IM, so patients with tumors with a high malignant potential should be offered IM as an adjuvant therapy. Preoperative IM can be given in cases of unresectable or borderline resectable cases. This might improve resectability.

## Conclusion

Duodenal GIST should be suspected in any patient with a duodenal wall mass. Extramural growth and central ulceration with or without bleeding should alert the endoscopist to the possibility of this diagnosis. There is more than one surgical approach available, but the absolute requirement is complete surgical excision. Preoperative IM can be considered in unresectable or borderline resectable cases.

## Consent

Written informed consent was obtained from the patient for publication of this case report and accompanying images. A copy of the written consent is available for review by the Editor-in-Chief of this journal.

## Competing interests

The authors declare that they have no competing interests.

## Authors' contributions

BM performed the literature review, collected the photos and wrote the article. FA collected some papers for review and provided input for the article. All authors read and approved the final manuscript.
